# Prevalence of Four Nematode Species (Mermithidae) in Adult Mosquitoes (Diptera: Culicidae): First Comments Since Infection/Parasitism in Fourth-Instar Larvae

**DOI:** 10.3390/microorganisms12122388

**Published:** 2024-11-21

**Authors:** Rafael Pérez-Pacheco, Edward G. Platzer, Carlos Granados-Echegoyen, Sabino H. Martinez-Tomas, Baldomero H. Zárate-Nicolás, Beatriz Quiroz-González, Henry Loeza-Concha, Jorge Tucuch-Haas, Alicia Fonseca-Muñoz, Fabián Arroyo-Balán

**Affiliations:** 1 Instituto Politécnico Nacional (IPN), Centro Interdisciplinario de Investigación para el Desarrollo Integral Regional (CIIDIR) Unidad Oaxaca, Santa Cruz Xoxocotlan 71230, Oaxaca, Mexico; smartinezt@ipn.mx (S.H.M.-T.); bzaraten@ipn.mx (B.H.Z.-N.); bquirozg@ipn.mx (B.Q.-G.); 2 University of California, Riverside, CA 92521, USA; edward.platzer@ucr.edu; 3 CONAHCYT-Instituto Politécnico Nacional (IPN), Centro Interdisciplinario de Investigación para el Desarrollo Integral Regional (CIIDIR) Unidad Oaxaca, Santa Cruz Xoxocotlan 71230, Oaxaca, Mexico; 4 Colegio de Postgraduados Campus Campeche, Champoton 24450, Campeche, Mexico; loeza.jesus@colpos.mx; 5 Instituto Nacional De Investigación Forestal, Agrícola y Pecuario, Mococha 97000, Yucatán, Mexico; leamsi182@hotmail.com; 6 Facultad de Sistemas Biológicos e Innovación Tecnológica, Universidad Autónoma Benito Juárez de Oaxaca, Oaxaca 68120, Oaxaca, Mexico; afonseca.cat@uabjo.mx; 7 Centro de Estudios en Desarrollo Sustentable y Aprovechamiento de la Vida Silvestre (CEDESU), CONAHCYT-Universidad Autónoma de Campeche, San Francisco de Campeche 24079, Campeche, Mexico; fabian.arroyo@conahcyt.mx

**Keywords:** nematodes, mosquito larvae, adult phase, prevalence of infection, infectivity

## Abstract

We examined the infective capacity of the mermithid nematodes, *Romanomermis iyengari*, *Romanomermis culicivorax*, *Romanomermis wuchangensis*, and *Strelkovimermis spiculatus* in fourth-instar mosquito larvae nearing pupation of *Aedes aegypti*, *Aedes sierrensis*, and *Culex pipiens* to determine their prevalence in the adults of these mosquitoes. We exposed **100** fourth-instar larvae to pre-parasitic nematodes (juvenile 2 stages) at a ratio of 10:1 (10 nematodes per mosquito larvae). Two days after the nematode applications, a sample of 20 pupae was taken and placed into transparent plastic cups with distilled water to observe the development and growth of pupae until they reached the adult phase with nematodes inside. The four species of nematodes showed the highest prevalence of infection on the *Cx. pipiens* mosquito, exceeding 55% parasitism, while *R. wuchangensis* and *R. iyengari* surpassed this value on *Ae. sierrensis* by 61.11% and 57.89%. *Aedes aegypti* was the least susceptible to nematodes, with parasitism values between 30% and 40%. In laboratory settings, we obtained high rates (26.32–77.78%) of parasitized adults when the three-mosquito species in fourth-instar larvae nearing pupation were exposed to infective nematodes. *R. wuchangensis* (1.86) and *S. spiculatus* (1.80) were infected *Cx. pipiens* with greater intensity and *R. iyengari* (1.33) and *R. culicivorax* (1.09) with less intensity. This evaluation offers valuable insights into the variability of nematode prevalence of infection and infectivity in fourth-instar larvae, which host mermithids capable of progressing through the pupal stage to adulthood.

## 1. Introduction

Mermithid nematodes have been identified in various mosquito species across the globe, with documented parasitism in at least 100 different species of mosquitoes [1].

There are many advantages to using mermithids for the biological control of mosquitoes, including host specificity, ease of application, and the lethality of a single nematode that successfully parasitizes an individual larva. Additionally, there is potential for recycling at long-term application sites, even in the extreme abiotic conditions found in certain tropical regions [2].

An alternative method for reducing mosquito populations is biological control, which involves the use of parasitic nematodes from the Mermithidae family. Several researchers have demonstrated the potential of *Romanomermis iyengari* (Welch, 1964) and *Romanomermis culicivorax* (Ross and Smith, 1976) (Nematoda: Mermithidae) nematodes for controlling mosquito larvae [3,4,5,6,7]. However, most studies focus on assessing the impact of nematodes on mosquito larvae during their first and second instar stages due to their susceptibility. The larvae parasitized by juvenile nematodes perish during the fourth mosquito larval stage when the adult nematode emerges to continue its biological cycle. Several studies have indicated that the third and fourth stages of mosquito larvae are less susceptible to nematode parasitism compared to the first and second instar stages [8,9,10,11,12,13,14]. Nonetheless, the third and fourth stages of mosquito larvae can still be parasitized, and nematode pre-parasites can continue their development into the pupal and adult stages. The emergence of Mermithidae nematodes invariably leads to the death of the mosquito, whether it is in the larval, pupal, or adult stage. This lethal effect on insects is crucial for public health due to the narrow host specificity, sparking significant interest in using mermithids for the biological control of mosquitoes.

Severe outbreaks occur because of the crucial role played by the *Culex pipiens* (Linnaeus, 1758) mosquito in transmitting diseases like St. Louis encephalitis and West Nile virus. Public health and prevention expenses spend millions of dollars to control these mosquitoes and manage the diseases they spread. Across different regions and outbreaks, the morbidity and mortality rates of *Cx. pipiens* can vary. For instance, the West Nile virus has a mortality rate of 1% in severe cases. In the United States, around 2000 cases of neuroinvasive disease were reported in 2018, with a mortality rate of 10% in severe cases [15,16]. *Aedes aegypti* (Linnaeus, 1762) mosquito is the primary vector of viral diseases such as dengue, Zika, and chikungunya. This mosquito causes between 96 and 390 million infections worldwide each year. In Mexico, dengue is one of the major diseases transmitted by *Ae. aegypti*, and its incidence has increased in recent decades. Between 2010 and 2015, the number of cases reported to the WHO rose from 2.2 million to 3.2 million. It is estimated that dengue poses a risk to almost half of the world’s population [17,18,19]. *Aedes sierrensis* (Ludlow, 1905) mosquito is less well-known and studied than other mosquitoes, but researchers have linked it to the transmission of diseases such as Sierra encephalitis virus, with no significant morbidity or mortality figures reported compared to more prominent vectors [20,21]. However, the possibility of symbiotic interactions between mosquitoes and mosquito viruses should not be ruled out [22].

The present study aims to assess the infective potential of four mosquito parasitic nematode species—*R. iyengari*, *R. culicivorax*, *R. wuchangensis* (BAO, 1985), and *Strelkovimermis spiculatus* (Poinar and Camino, 1986)—on fourth-instar larvae approaching the pupation of *Ae. aegypti*, *Ae. sierrensis*, and *Cx. pipiens* (Diptera: Culicidae) to investigate the biological development of nematodes into the pupal and adult stages of the mosquito species in this study.

## 2. Materials and Methods

### 2.1. Nematode Colony

The nematodes *R. culicivorax*, *R. iyengari*, *R. wuchangensis*, and *S. spiculatus* were reared using second instar larvae of the mosquito Culex pipiens, following the methods outlined by Petersen and Willis [23] and Pérez-Pacheco et al. [5] at the University of California, Riverside. Mosquito egg packets were placed in plastic trays measuring 47 × 35 × 12 cm, which contained 4 L of distilled water.

Pre-parasitic nematode larvae (L2 stage) were obtained from cultures that had been stored in the laboratory for six weeks. Distilled water was added to these cultures, and after 3 to 4 h, the water was decanted into an Erlenmeyer flask. The volume required for infestation was then calculated using a stereoscopic microscope and the volumetric dilution method.

L2 nematodes were applied at a ratio of 5:1 (five nematodes per mosquito larva) when the larvae reached the second instar; a 5:1 ratio was employed to maintain the nematode brood stock, which differs from the ratio utilized in the experimental process. After 3 to 6 days of exposure, the larvae reached the fourth instar, and dead larvae were observed floating on the surface of the water. These were then sieved to collect the adult nematodes emerging from the host larvae.

The emerged post-parasitic nematodes were placed in containers measuring 21 × 13.5 × 5.5 cm using curved-tip entomological needles. They were mixed with previously washed and sterilized river sand along with 50 mL of distilled water. This environment facilitated the colonization of the nematodes at the bottom of the container and promoted their mating and oviposition on the substrate. The water was subsequently decanted, and the containers were hermetically sealed to prevent desiccation of the culture. The cultures were labeled by species and planting date and stored at room temperature (27 ± 2 °C) for six weeks.

### 2.2. Mosquito Colony

*Culex* pipiens mosquitoes were bred at the University of California, Riverside. *Aedes sierrensis* eggs were collected using ovitraps in the Lake County Vector Control District, California. *Aedes aegypti* eggs were obtained from a colony maintained by Dr. Peter Atkinson in the Department of Entomology at UC Riverside. All mosquitoes were reared in the laboratory. The larvae were fed tilapia fish food (Api-tilapia-1^®^) until they reached the pupal stage, at which point they were transferred to containers measuring 47 cm × 35 cm × 12 cm filled with distilled water and placed in insect cages to develop into adults.

Adult mosquitoes were given ad libitum access to a 10% sugar solution. Every 12 h, a sedated rabbit (*Oryctolagus cuniculus*) was provided as a blood meal for the female mosquitoes. To facilitate egg-laying, black plastic containers (30 × 20 × 6 cm) with Pellon^®^ fabric attached to the walls were placed in the cages, allowing gravid females to lay their eggs. The collected egg-laying material, including egg rafts from the Culex mosquitoes (which typically contained 100 to 200 eggs), was dried for later use.

The breeding environment was maintained at 27 ± 2 °C, with a relative humidity of 60–70% and a 12 h light/dark photoperiod. The F1 generation of mosquitoes produced in this environment was utilized in a bioassay [24,25].

### 2.3. Adult Parasitism

One hundred late fourth-instar mosquito larvae were introduced into 500 mL of distilled water in polyethylene trays measuring 21 cm in length, 13.5 cm in width, and 5.5 cm in height at a parasite–host ratio of 10:1 (10 nematodes per larva) and replicated four times (n = 400). Two days after exposure to pre-parasitic nematodes, 20 larvae were randomly sampled from each replicate (n = 80) and transferred into clear plastic cups containing 150 mL of distilled water. The cups were covered with plastic plugs featuring 5 mm perforations to allow for adequate oxygen access. From this sample, we observed the daily development of mosquito larvae and removed deceased individuals for microscopic dissection to assess their parasitic status, using a Zeiss Stemi DV4, 8–32X stereo microscope (Jena, Germany). As some of the larvae matured into adults, we examined the adult mosquitoes for nematodes. The prevalence of infection (%) and the intensity of infection of four species of nematodes (the number of nematodes per adult mosquito) were determined by counting the number of parasitized mosquitoes and the number of nematodes that emerged from mosquitoes. The environmental conditions during the parasitism experiments were set to a temperature of 27 ± 2 °C, with relative humidity between 60% and 70%, and a 12 h light/dark cycle. A control group was included in which the mosquito larvae were not exposed to the nematode dosage but were subjected to the same environmental conditions.

### 2.4. Statistical Analysis

The experiment was conducted using a completely randomized experimental design with four repetitions. Analysis of variance was employed to identify significant differences, and Tukey’s test was utilized to distinguish means at a significance level of *p* < 0.05. In the experiment, a sample of 20 individuals per 100 larvae was considered for each replicate because this population per replicate is not extremely heterogeneous (the 100 individuals are relatively homogeneous in terms of age, size, and health). In addition, random sampling (n = 20/replicate) ensured that each individual had the same probability of being selected, which increased the representativeness of the sample [26]. Furthermore, in populations of moderate size, such as 100 larvae per replicate, a sample of 20 individuals is usually sufficient to estimate the prevalence of a phenomenon (such as nematode infection) with an acceptable level of precision [27], as shown in previous studies [5,28].

## 3. Results and Discussion

Understanding whether nematodes can emerge from adult mosquitoes is essential for clarifying their life cycle. This knowledge is crucial for developing effective control and management strategies, as some nematode species may have additional life stages in adult mosquitoes that are not apparent when observing larvae alone. Overall, *Cx. pipiens* and *Ae. sierrensis* exhibited the highest levels of infection. *Romanomermis wuchangensis* caused the highest rates of parasitism in *Cx. pipiens* and *Ae. sierrensis*, resulting in parasitism percentages of 77.78% and 61.11%, respectively.

Similarly, *R. iyengari* caused parasitism percentages of 66.67% in *Cx. pipiens* and 57.89% in *Ae. Sierrensis*. The nematode *S. spiculatus* induced 40% parasitism in *Ae. Aegypti,* which was the least susceptible among the four mosquito species. High rates (26.32–77.78%) of parasitized adults were obtained in laboratory settings when the three-mosquito species in fourth-instar larvae nearing pupation were exposed to infective nematodes (Table 1).

*Culex pipiens* exhibited higher infection intensity when exposed to *R. wuchangensis* (1.86) and *S. spiculatus* (1.80), and lower intensity when infected by *R. iyengari* (1.33) and *R. culicivorax* (1.09). For *Ae. sierrensis*, the highest infection intensities were reported for *R. iyengari* (1.91) and *S. spiculatus* (1.60), and lower intensities with *R. wuchangensis* (1.27). *R. culicivorax* had an intensity of 1.14. *Aedes aegypti* showed lower levels of infection intensity, with no statistically significant difference observed in the infection caused by the four-nematode species (Table 2).

The mortality of adult mosquitoes that emerged from pupae parasitized during the fourth instar larvae stage was observed between the fourth and eighth day. This period marks the end of the parasitic phase of the nematodes, prompting their emergence to proceed with their life cycle. It was observed and verified that the evolution of the parasitic phase of the nematode normally occurs through the pupal and the adult phases of the mosquito, meaning when they are infested in the fourth instar. This implies that the life expectancy of the adult is drastically reduced, preventing it from becoming a vector of diseases (e.g., *Ae. aegypti* for Dengue Fever, Chikungunya, Zika Virus, Yellow Fever; *Ae. sierrensis* for Western Equine Encephalitis, California Encephalitis; and *Cx. pipiens* for West Nile Virus, St. Louis Encephalitis, and Japanese Encephalitis). The reason is that during this short lifespan of the pathogen, the disease cannot be incubated into the adult phase of the mosquito.

The outcome of parasitism by different nematode species in mosquito larvae is influenced by several major factors. These include the high chitin content of the mosquito larvae’s exoskeleton [29], the distinct movement patterns of the larvae of each nematode species [30], and the unique physiological characteristics of the mosquito larvae [31]. These factors are more prominent in fourth-instar mosquito larvae compared to first-instar mosquito larvae. Furthermore, late-fourth instar mosquito larvae, which are nearing the pupal stage, have reduced exposure to circulating nematodes as they progress more quickly to the pupal stage, decreasing the likelihood of parasitism. At this stage, larvae are more developed and exhibit faster movement compared to second-instar larvae, which are commonly identified as hosts of nematodes in mosquitoes. The insights from this study provide valuable information to enhance the effectiveness of using this biological control method for managing mosquitoes in natural conditions.

According to Allahverdipoura et al. [32], there is a correlation between the duration of parasite infection in fourth-instar larvae before transitioning to the pupal stage and the probability of detecting mermithids in adult mosquitoes. They also point out that exposure to early fourth-instar larvae does not result in parasitized adults. In contrast, Kurihara and Maeda [33] observed that exposure of three-day-old fourth instar larvae of *Culex pipiens molestus* to the nematode *R. culicivorax* did not produce parasitized adults. However, exposure to four-day-old fourth-instar larvae resulted in the parasitism of adults.

**Figure 1:**
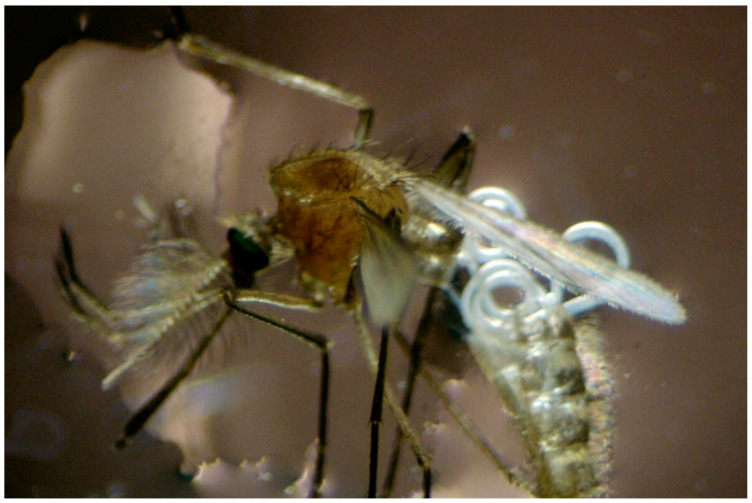
*Culex pipiens* adult mosquito with a distended, abdomen from emerging the parasitic mermithids nematode *R. wuchangensis*.

There is currently no recent information on the occurrence of parasitic nematodes in adult mosquitoes. This is also the first report of the nematode *R. wuchangensis* occurring and emerging from adult *Cx. pipiens* mosquitoes. However, parasitized adult mosquitoes could potentially serve as a way for mermithids to establish themselves in new host larval habitats. Mermithids need to locate an aquatic mosquito larva to complete their life cycle, as water is the only habitat for their free-living stages (Figure 1).

## 4. Conclusions

This study demonstrates that the four species of mermithid nematodes (*R. iyengari*, *R. culicivorax*, *R. wuchangensis*, and *S. spiculatus*) can infect fourth instar larvae of *Cx*. *pipiens*, *Ae. sierrensis*, and *Ae. aegypti*, with some nematodes continuing their life cycle to the adult mosquito stage. The species *R. wuchangensis* and *R. iyengari* exhibited the highest prevalence levels of infection in *Cx. pipiens* and *Ae. sierrensis*, exceeding 65%. In contrast, *Ae. aegypti* was consistently less susceptible to infection by all evaluated nematode species.

Furthermore, in terms of infectivity, *R. wuchangensis* and *S. spiculatus* demonstrated the highest levels of infectivity in *Cx. pipiens* and *Ae. sierrensis*, surpassing the other nematode species in these two mosquito species. This evaluation provides valuable information about the variability in the infective capacity of nematodes in the fourth instar larvae stage, supporting the idea that mermithids can pass through the pupal stage to the adult stage. These results highlight the variability in susceptibility among different mosquito species to these nematodes and their potential in the implementation of biological control strategies. The high parasitism rates achieved under laboratory conditions suggest that these nematodes could serve as an effective tool for mosquito control in the field, contributing to the reduction in populations of disease vectors.

## Figures and Tables

**Table 1:** Prevalence of infection by four species of nematodes in adult mosquitoes (Cx. *pipiens*, Ae. *Sierrensis*, and *Ae. Aegypti*).

Nematode	Prevalence of Infection (%)/Mosquito Species			*p*-Value *
	*Culex pipiens*	*Aedes sierrensis*	*Aedes aegypti*	
*R. wuchangensis*	77.78 A a	61.11 A a	30.00 A b	<0.001
*R. iyengari*	66.67 AB a	57.89 A a	31.58 A b	0.005
*R. culicivorax*	55.00 B a	33.33 B b	33.33 A b	0.0217
*S. spiculatus*	55.56 B a	26.32 B b	40.00 A ab	0.0068
*Control*	0.00 C c	0.00 C c	0.00 C c	-
*p*-value **	0.0348	<0.0001	0.6751	

* Chi-square test *p*-value between mosquitos. ** Chi-square test *p*-value between nematodes. Uppercase letters by columns denote a statistical difference between nematodes. Lowercase letters by rows denote a statistical difference between mosquitos’ species (n = 20 mosquitos/nematode species).

**Table 2:** Infection intensity of four species of nematodes in adult mosquitoes *Cx. pipiens, Ae. sierrensis*, and *Ae. aegypti*.

Nematode	Infectivity/Mosquito Species			*p*-Value *
	*Culex pipiens*	*Aedes sierrensis*	*Aedes aegypti*	
*R. wuchangensis*	1.86 A a	1.27 BC b	1.33 A b	0.0015
*S. spiculatus*	1.80 A a	1.60 AB a	1.13 A b	0.0012
*R. iyengari*	1.33 B b	1.91 A a	1.17 A b	0.0367
*R. culicivorax*	1.09 B b	1.14 C ab	1.50 A a	0.0008
*p*-value **	<0.0001	0.0003	0.1251	

* Chi-square test *p*-value between mosquitos. ** Chi-square test *p*-value between nematodes. Uppercase letters by columns denote a statistical difference between nematodes. Lowercase letters by rows denote a statistical difference between mosquitos’ species.

## Data Availability

The original contributions presented in the study are included in the article, further inquiries can be directed to the corresponding authors.
